# Significance of Mannose-Binding Lectin Deficiency and Nucleotide-Binding Oligomerization Domain 2 Polymorphisms in *Staphylococcus aureus* Bloodstream Infections: A Case-Control Study

**DOI:** 10.1371/journal.pone.0076218

**Published:** 2013-09-27

**Authors:** Michael Osthoff, Hue Mun Au Yong, Melinda M. Dean, Damon P. Eisen

**Affiliations:** 1 Victorian Infectious Diseases Service, Royal Melbourne Hospital, Parkville, Victoria, Australia; 2 Department of Medicine, Royal Melbourne Hospital, University of Melbourne, Victoria, Australia; 3 Research and Development, Australian Red Cross Blood Service, Queensland, Australia; South Texas Veterans Health Care System and University Health Science Center San Antonio, United States of America

## Abstract

**Background:**

Pathways coordinated by innate pattern recognition receptors like mannose-binding lectin (MBL) and nucleotide-binding oligomerization domain 2 (NOD2) are among the first immune responses to *Staphylococcus aureus* (*S. aureus*) bloodstream infections (BSI) in animal models, but human data are limited. Here, we investigated the role of MBL deficiency and *NOD2* mutations in the predisposition to and severity of *S. aureus* BSI.

**Patients and Methods:**

A matched case-control study was undertaken involving 70 patients with *S. aureus* BSI and 70 age- and sex-matched hospitalized controls. MBL levels, *MBL2* and *NOD2* polymorphisms were analyzed.

**Results:**

After adjusting for potential confounders, MBL deficiency (<0.5 µg/ml) was found less frequently in cases than controls (26 vs. 41%, OR 0.4, 95% confidence interval (CI) 0.20-0.95, p=0.04) as were low producing MBL genotypes (11 vs. 23%, OR 0.2, 95% CI 0.08-0.75, p=0.01), whereas *NOD2* polymorphisms were similarly distributed. Cases with *NOD2* polymorphisms had less organ dysfunction as shown by a lower SOFA score (median 2.5 vs. 4.5, p=0.02), whereas only severe MBL deficiency (<0.1 µg/ml) was associated with life-threatening *S. aureus* BSI (OR 5.6, 95% CI 1.25-24.85, p=0.02).

**Conclusions:**

Contrary to animal model data, our study suggests MBL deficiency may confer protection against acquiring *S. aureus* BSI. *NOD2* mutations were less frequently associated with multi-organ dysfunction. Further human studies of the innate immune response in *S. aureus* BSI are needed to identify suitable host targets in sepsis treatment.

## Introduction

*Staphylococcus aureus* (*S. aureus*) is a major cause of nosocomial and community-acquired bloodstream infections (BSI) accounting for up to 20% of hospital isolates [1]. *S. aureus* BSI is associated with a high morbidity and mortality compared to other BSI pathogens [2] and when it is caused by methicillin resistant isolates the mortality is even greater [3]. These infections place a huge burden on health care systems due to a longer duration of hospital stay and higher total treatment cost compared to bacteremia caused by any other pathogen [4]. In addition, the incidence of *S. aureus* BSI has steadily increased over the past 30 years as a consequence of frequent use of intravascular devices and invasive procedures [5]. General host risk factors for the acquisition of *S. aureus* BSI include staphylococcal colonization, surgical site infection, injection drug use, presence of immunosuppressive conditions and liver disease [2]. Central to the pathogenicity and immune evasion of *S. aureus* is the coordinated activity of several virulence factors including surface-expressed adhesins, complement inhibitors, exotoxins and exoenzymes that facilitate direct tissue destruction while avoiding activation of the innate immune system, particularly the complement system [6]. However, human studies examining the impact of the innate immune system on the susceptibility to and the severity of *S. aureus* BSI are limited [7,8].

Pattern recognition receptors (PRR) are crucially involved in the initial and immediate immune response against *S. aureus* (reviewed in [9]). In particular, nucleotide-binding oligomerization domain 2 (NOD2) and mannose-binding lectin (MBL) have been implicated in the pathogenesis of *S. aureus* infections in several experimental models. NOD2 is an intracellular sensor for both gram-positive and -negative bacterial cell wall components leading to a pro-inflammatory NF-κB and IL-1β mediated cytokine response (reviewed in [10]), although the exact mechanism and regulation of response in bacterial infections still remain to be fully elucidated. Animal model data on *S. aureus* and NOD2 are conflicting [11–13]. Results from two studies involving critically-ill sepsis patients suggest an increased risk of bacteremia and mortality in individuals with at least one NOD2 variant [14,15].

MBL, a liver-derived circulating lectin contributes to the efficient removal of pathogens and apoptotic cells by activating the lectin pathway of complement and promoting opsonophagocytosis [16], and has been implicated as an important defense mechanism in various infectious diseases [17]. Functional MBL deficiency is common in humans and is caused by polymorphisms within the coding and promoter regions of the *MBL2* gene on chromosome 10 [18]. In vitro, MBL is able to bind to *S. aureus* [19] and evidence from animal models suggests that MBL deficiency significantly increases the susceptibility to and severity of *S. aureus* bacteremia [20,21]. However, its contribution to *S. aureus* induced complement activation and phagocytosis of *S. aureus* in adults is probably less than the antibody-mediated classical pathway activation [22–24]. Several clinical studies have reported a correlation between MBL deficiency and increased susceptibility to bacterial sepsis in children and adults [25–27].

Given these data on the potential role of NOD2 and MBL in human innate immune defences against severe *S. aureus* infection we hypothesized that MBL deficiency and NOD2 mutations might be associated with increased susceptibility to and severity of *S. aureus* BSI.

## Patients and Methods

### Ethics statement

The study had been approved by the Melbourne Health Human Research and Ethics Committee and all participants gave written informed consent for the study.

### Participants

We conducted a matched prospective case-control study at two major tertiary hospitals involving 70 patients with *S. aureus* BSI and 70 age- and sex-matched hospitalized controls. Investigators were notified of all blood cultures positive for *S. aureus* by the central microbiology laboratory during the study period (September 2009 to September 2011). Case patients were enrolled with their first *S. aureus* BSI if they were >18 years old and had at least 1 positive blood culture for *S. aureus*. Hospitalised control patients were selected on the absence of infection as the cause for admission and were matched for age (within 2 years) and sex. Controls had to be admitted within 2 months of the case patient. To increase the power for the analysis of severity after *S. aureus* BSI, 30 patients with *S. aureus* BSI with similar epidemiology from a previous study were included only in this component of the study [25]. MBL levels and *MBL2* genotype have been previously reported for these patients, and demographic and clinical data similar to the patients recruited in this study was available. We were able to use stored genomic DNA samples from these patients to determine *NOD2* polymorphisms.

### Risk factors for staphylococcal BSI

Demographic, clinical and microbiological data were collected by investigators blinded to MBL and/or NOD2 results including comorbidities and presence of intravenous (IV) lines or urinary catheters before the episode of *S. aureus* BSI. Liver disease was defined as cirrhosis, chronic hepatitis B and C, hepatocellular carcinoma or any other significant acute or chronic liver disease. Renal disease included acute and chronic renal impairment of various reasons excluding hemodialysis. Patients were regarded as immunosuppressed if they were receiving chemotherapy, corticosteroids (>7.5mg prednisolone equivalent per day), methotrexate, cyclosporine, tacrolimus, azathioprine or biologics such as TNF-α inhibitors.

The Sequential Organ Failure Assessment (SOFA) score was calculated for case patients on the day when the first positive blood culture was taken. A SOFA score of >7 was regarded as very severe disease being the mean score of non-survivors in the validation study of this score [28].

### Determination of MBL plasma levels

EDTA blood samples which had been taken one to three days prior to the diagnosis of *S. aureus* BSI were accessed for further testing. Quantification of MBL plasma levels was performed by an investigator blinded to any patient data using a mannan-binding enzyme-linked immunosorbent assay as previously described [25,29]. Briefly, mannan-coated microtitre plates were incubated with samples at 1:25 and 1:100 dilutions for 90 min at room temperature followed by detection of bound MBL with a biotinylated monoclonal anti-MBL antibody (HYB 131-01, BioPorto Diagnostics, Denmark). MBL deficiency was defined as serum level < 0.5 µg/ml and, severe as < 0.1 µg/ml, respectively.

### MBL2 and NOD2 genotyping

*MBL2* promoter and first exon and *NOD2* polymorphisms were determined by allele specific polymerase chain reaction (PCR) using TaqMan fluorescent probes (TaqMan genotyping assays, Life Technologies, Australia). For assay details, see Table S1. DNA lysates were prepared from 2µl of stored buffy coat according to the manufacturer’s instruction (TaqMan Sample-to-SNP, Life Technologies, Australia), and stored genomic DNA was used for 30 patients included in a previous study [25]. For all TaqMan assays, DNA amplification was carried out in 5µL volume reactions containing 1µl of DNA lysate or 20ng of genomic DNA, 0.25µl TaqMan genotyping assay mix, 2.5µl TaqMan GTXpress Master Mix (Life Technologies, Australia) and 1.25µl DNase-free water. All reactions were performed in 384-well plates and in the ViiA 7 thermocycler (Life Technologies, Australia) according to the manufacturer’s instructions. For allelic discrimination end-point fluorescence was read at 25°C, and the ViiA 7 software was used to analyze the results (Life Technologies, Australia).

*MBL2* genotypes were classified as low (*XA/YO, YO/YO*), intermediate (*XA/XA, YA/YO*) or high (*YA/YA, XA/YA*) producing genotypes according to published literature [26] with exon variant alleles collectively designated as *O* and the wild-type gene as *A*, and the promoter variant allele and the wild-type gene designated as *X* and *Y*, respectively.

### Definition of aims

The main aim of this study used to determine the sample size, was to compare the frequency of MBL deficiency in patients with *S. aureus* BSI with age/sex-matched, hospitalized control patients. We recruited 70 cases and controls in order to have an 80% chance of detecting an odds ratio of 3, with an expected frequency of MBL deficiency (defined as plasma concentration <0.5 µg/ml) in the control population of 24% [29] at the 5% level of significance. Additional aims included measuring the effect of *NOD2* mutations on the risk of acquiring *S. aureus* BSI in cases compared to controls, and in cases alone, the influence of MBL levels and *MBL2* and *NOD2* mutations on the severity of *S. aureus* BSI as evaluated by the SOFA score and crude in-hospital mortality.

### Statistical analysis

To investigate potential risk factors for acquiring *S. aureus* BSI, matched univariate analysis was performed by running conditional logistic regression on one variable at a time with *S. aureus* BSI as the dependent variable. In addition, Wilcoxon signed-rank test was applied to compare MBL levels in cases and matched controls. Multivariate conditional logistic regression models were used to estimate the effect of MBL deficiency on the risk of acquiring *S. aureus* BSI while adjusting for covariables with univariate p values less than 0.1 and which have been described in previous studies.

Regarding the severity of *S. aureus* BSI differences in outcome measures of cases according to patient characteristics, MBL levels and *MBL2* or *NOD2* mutations were first analyzed using the Fisher’s exact, the χ^2^ or the Mann-Whitney-U-Test where appropriate. Subsequently, stepwise binary logistic regression models were calculated to estimate the association of MBL levels and *MBL2* or *NOD2* mutations with predefined endpoints in multivariate analyses after adjustment for covariables with univariate p values less than 0.1. The Hardy-Weinberg equilibrium for *MBL2* and *NOD2* genotype frequencies was assessed by χ^2^ statistics. All testing was two-tailed. All analyses were performed using SPSS statistics, version 17.0 (SPSS Inc., USA).

## Results

### Demographic and clinical characteristics of cases and controls

The analyzed study population consisted of 70 *S. aureus* BSI cases and 70 age- and sex-matched, hospitalized controls. *S. aureus* BSI were nosocomially acquired (60%) and related to endovascular sources (49%) in the majority of cases. Controls were mainly admitted for trauma or elective surgery (Table 1). A median of 2 blood culture bottles were positive for *S. aureus*, and cultured isolates were methicillin resistant in 12/70 (17%) of cases (2/12 community-acquired). Antibiotic therapy appropriate for the susceptibility of the infective organism was received within 24 hours after blood cultures had been drawn in 59/70 (84%) cases.

**Table 1:** Clinical characteristics of *S. aureus* BSI cases and controls.

	**Cases (n=70)**
	**n (%)**
**Infective source**	
Intravascular line	16 (23)
Skin or soft tissue	11 (16)
Bone or joint	13 (19)
Endocarditis	7 (10)
Pneumonia	8 (11)
Hemodialysis associated	11 (16)
No source identified	4 (6)
**Microbiology**	
PSSA	15 (21)
MRSA	12 (17)
	**Controls (n=70)**
	**n (%)**
**Admission diagnosis, n (%)**	
Trauma	18 (26)
Medical condition	29 (41)
Surgery (not trauma related)	23 (33)

Abbreviations: BSI, bloodstream infection; MRSA, methicillin-resistant *S. aureus*; PSSA, penicillin-sensitive *S. aureus*; *S. aureus*, *Staphylococcus aureus*.

In terms of risk factors, *S. aureus* BSI cases were more likely to suffer from liver disease, to require hemodialysis and to have long-term IV lines when compared to controls (Table 2). In contrast, controls were more likely to have undergone recent surgery, with peripheral IV lines and urinary catheters used more frequently at the time of recruitment. There was no difference in terms of prevalence of diabetes mellitus, heart disease, cancer or immunosuppression.

**Table 2:** Analysis of clinical characteristics as predisposing risk factors for *S. aureus* BSI.

**Variables**	**Controls**	**Cases**	**Univariate matched analysis**	
	(n=70)	(n=70)	OD (95% CI) P value^a^	
Age, mean (SD)	61 (17)	61 (18)	1.09 (0.9-1.32)	0.4
Male sex, n (%)	49 (70)	51 (73)	65 (0.01-5x10^6^)	0.45
Diabetes, n (%)	15 (21)	20 (29)	1.63 (0.67-3.92)	0.28
Heart disease, n (%)	36 (51)	30 (43)	0.46 (0.16-1.31)	0.14
Cancer, n (%)	10 (14)	13 (19)	1.36 (0.55-3.42)	0.5
Immunosuppressive treatment n (%)	8 (11)	15 (21)	2.2 (0.82-5.70)	0.12
Liver disease, n (%)	0 (0)	15 (21)	65 (1.03-4148.5)	**<0.05**
Kidney disease, n (%)	6 (9)	10 (14)	2.0 (0.60-6.64)	0.26
Hemodialysis, n (%)	3 (4)	16 (23)	5.33 (1.55-18.3)	**<0.01**
Illicit IV drug use, n (%)	2 (3)	6 (9)	65 (0.02-2x10^5^)	0.31
Recent surgery, n (%)	41 (59)	7 (10)	0.08 (0.03-0.26)	**<0.001**
Urinary catheter, n (%)	30 (43)	9 (13)	0.22 (0.09-0.54)	**0.001**
Vascular lines, n (%)				
Long-term IV line	14 (20)	28 (40)	2.1 (1.1-4.0)	**0.03**
Peripheral IV line	57 (81)	26 (37)	0.11 (0.04-0.32)	**<0.001**

Abbreviations: BSI, bloodstream infection; CI, confidence interval; IQR, interquartile range; IV, intravenous; OD, odds ratio; SD, standard deviation; *S. aureus*, *Staphylococcus aureus*. Long-term IV line refers to a central, tunneled or peripherally inserted central intravenous catheter.

^a^ univariate conditional logistic regression analysis.

### Association of MBL and NOD2 variants with the risk of acquiring *S. aureus* BSI

**Figure 1:**
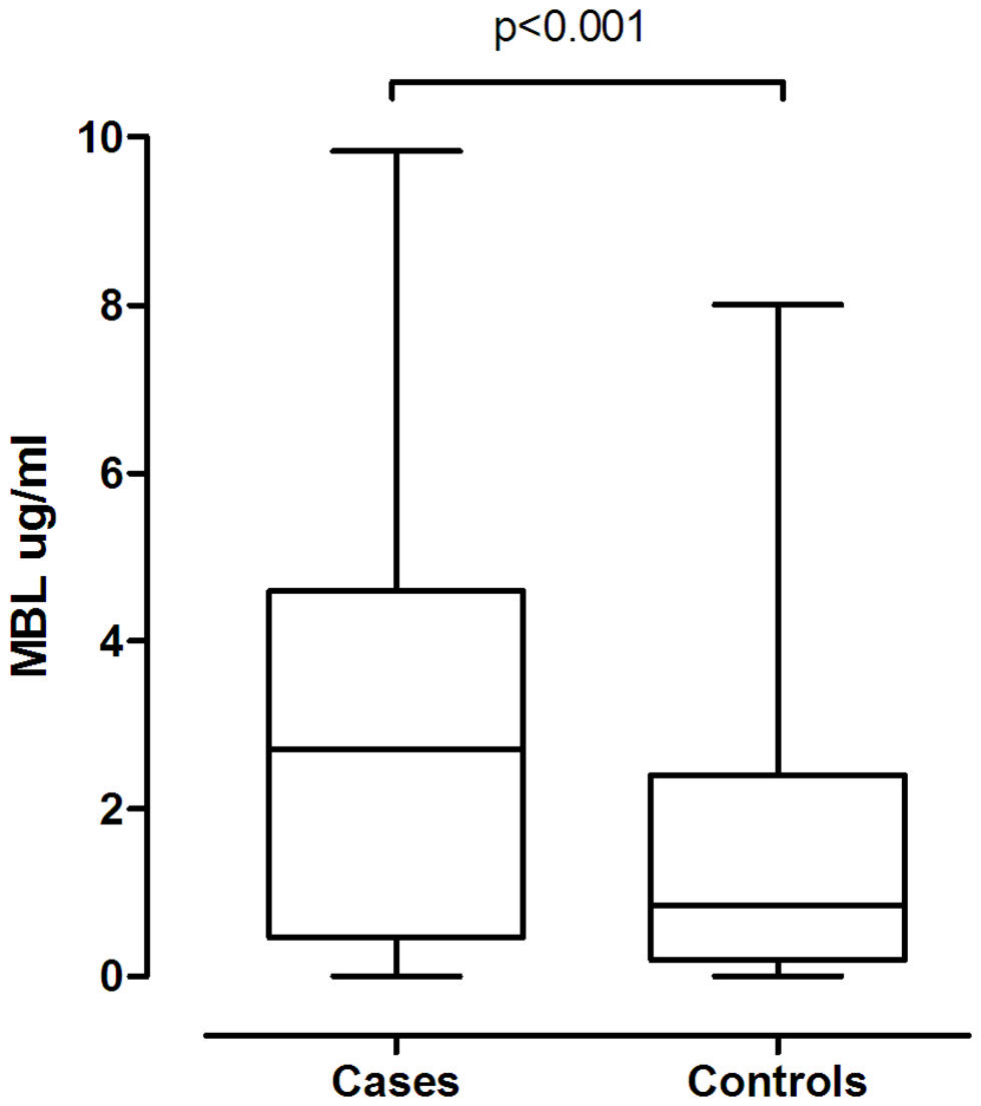
Plasma mannose-binding lectin levels in *S. aureus* BSI cases and controls. Differences in plasma mannose-binding lectin levels in *S. aureus* BSI cases and controls. Short horizontal lines (whiskers) represent minimum and maximum levels whereas horizontal lines inside the box-plot represent medians. Abbreviations: BSI, bloodstream infection; MBL, mannose-binding lectin. *S. aureus*, *Staphylococcus aureus*.

*MBL2* and *NOD2* allele frequencies at all 7 positions were in agreement with the predicted Hardy-Weinberg equilibrium (data not shown). There was significant correlation between *MBL2* genotypes and MBL levels (Kruskal-Wallis test, p<0.001, data not shown). As expected, patients with low producing genotypes all had MBL levels <0.5 µg/ml. The frequency distribution of *MBL2* genotypes differed significantly among cases and controls with higher number of intermediate and low genotypes in controls (Table 3). In line with *MBL2* genotypes, median MBL levels were significantly lower in controls compared to cases (0.9 (IQR 0.2-2.4) vs. 2.7 (IQR 0.5-4.6) µg/ml, p<0.001, Figure 1), and 18/70 of cases vs. 29/70 of controls had MBL levels <0.5 µg/ml. *NOD2* mutations were exclusively heterozygous and were found in 10/70 and seven-seventieths of cases and controls, respectively (p=0.4). Only three individuals had both low producing *MBL2* genotypes and a *NOD2* mutation (2 cases, 1 control).

**Table 3:** Analysis of *MBL2* and *NOD2* genotypes in *S. aureus* BSI cases and controls.

**Variables**	**Controls**	**Cases**	**Univariate matched analysis**	
	(n=70)	(n=70)	OD (95% CI) P value^a^	
*MBL2* exon variants, n (%)				
A/A	30 (43)	44 (63)	Reference	
A/B	22	16		
A/C	5	1		
A/D	8	5		
Total A/O	35 (50)	22 (31)	0.44 (0.22-0.90)	**0.024**
B/B	2	1		
B/C	1	1		
B/D	1	1		
C/D	1	0		
Total O/O	5 (7)	4 (6)	0.42 (0.10-1.79)	0.24
*MBL2* promoter variants, n (%)				
Y/Y	42 (60)	52 (74)	Reference	
Y/X	25 (36)	17 (24)	0.47 (0.20-1.12)	0.09
X/X	3 (4)	1 (1)	0.27 (0.03-2.70)	0.27
*MBL2* genotypes, n (%)				
YA/YA	13	30		
XA/YA	14	13		
Total high producing	27 (39)	43 (61)	Reference	
XA/XA	3	1		
YA/YO	24	18		
Total intermediate producing	27 (39)	19 (27)	0.43 (0.20-0.94)	**0.034**
XA/YO	11	4		
YO/YO	5	4		
Total low producing	16 (23)	8 (11)	0.31 (0.11-0.84)	**0.021**
MBL levels (µg/ml), median (IQR)	0.9 (0.2-2.4)	2.7 (0.5-4.6)	1.32 (1.10-1.58)^b^	**0.002**
MBL <0.5µg/ml, n (%)	29 (41)	18 (26)	0.50 (0.24-1.03)	0.06
*NOD2* mutations, n (%)	7 (10)	10 (14)	1.5 (0.53-4.21)	0.44
R702W C>T	4	4		
G908R G>C	2	2		
L1007fsinsC -/C	1	4		

Abbreviations: BSI, bloodstream infection; CI, confidence interval; IQR, interquartile range; MBL, mannose-binding lectin; NOD2, nucleotide-binding oligomerization domain 2; OD, odds ratio; SD, standard deviation; *S. aureus*, *Staphylococcus* *aureus*. Y and A denote *MBL2* promoter and exon wildtype alleles, respectively.

^a^ univariate conditional logistic regression analysis. ^b^ per 1 µg/ml increase in MBL serum levels.

Contrary to our *a priori* hypothesis, MBL deficiency defined by low producing genotypes or lower MBL plasma levels (continuous variable) along with recent surgery, indwelling urinary catheters, and peripheral IV lines were associated with protection from *S. aureus* BSI. Previously described factors were associated with increased risk of *S. aureus* BSI (Tables 2 and 3).

Three risk factors that were highly correlated with protection from *S. aureus* BSI were not included in the multivariate model (recent surgery, indwelling urinary catheter and peripheral IV line) as their associations were in contrast with current literature and likely related to selection bias in the control group (trauma or elective surgical patients were more likely to be recruited as controls with urinary and peripheral IV catheters more frequently present in those patients at recruitment). After adjusting for potential confounders MBL deficiency as defined by MBL levels <0.5 µg/ml (OR 0.44, 95% confidence interval (CI) 0.20-0.95, p=0.04) or low producing genotypes (OR 0.24, 95% CI 0.08-0.75, p=0.01) remained independently associated with a decreased risk of acquiring a *S. aureus* BSI. Otherwise, only hemodialysis was an independent predictor of *S. aureus* BSI in a multivariate analysis (OR 6.01, 95% CI 1.70-21.54, p<0.01).

### Association of MBL and NOD2 variants with severity of *S. aureus* BSI

One hundred cases were analyzed for associations between *MBL2* or *NOD2* mutations and severity of *S. aureus*. Clinical characteristics are displayed in Table 4. Regarding severity of *S. aureus* BSI the median SOFA score was 4 (IQR 2-6) and in-hospital mortality was 10% overall.

**Table 4:** Clinical characteristics and outcomes in *S. aureus* BSI cases (n=100).

**Variables**	***S. aureus* BSI**	**SOFA score**			
	(n=100)	<2 (n=32)	3-7 (n=52)	>7 (n=16)	P value
Age, mean (SD)	60.7 (18.7)	63.7 (18.5)	59.5 (19.0)	58.5 (18.8)	0.54
Male sex, n (%)	72	26 (81)	38 (73)	8 (50)	0.07
*MBL2* genotypes, n (%)					
high (YA/YA, XA/YA)	60	18 (56)	33 (64)	9 (56)	0.84
intermediate (YA/YO, XA/XA)	29	9 (28)	15 (29)	5 (31)	
low (YO/YO, XA/YO)	11	5 (16)	4 (8)	2 (13)	
MBL levels (µg/ml), median(IQR)	2.4 (0.5-4.1)	3.0 (0.7-6.0)	2.4 (0.5-3.6)	1.0 (0.1-4.0)	0.38
MBL <0.5µg/ml, n (%)	25	7 (22)	13 (25)	5 (31)	0.78
MBL <0.1µg/ml, n (%)	10	4 (13)	2 (4)	4 (25)	**0.04**
*NOD2* mutation, n (%)	16	8 (25)	7 (14)	1 (6)	0.19
Outcomes					
SOFA score, median (IQR)	4 (2-6)				
ICU admission, n (%)	22	1 (3)	11 (21)	10 (63)	**<0.001**
In-hospital mortality, n (%)	10	2 (6)	5 (10)	3 (19)	0.4

Abbreviations: BSI, bloodstream infection; CI, confidence interval; ICU, intensive care unit; IQR, interquartile range; MBL, mannose-binding lectin; NOD2, nucleotide-binding oligomerization domain 2; OD, odds ratio; SD, standard deviation; *S. aureus*, *Staphylococcus aureus*; SOFA, sequential organ failure assessment; Y and A denote *MBL2* promoter and exon wild type alleles respectively.

Organ dysfunction was less pronounced in patients with *NOD2* mutations indicated by significantly lower SOFA scores (median 2.5 (IQR 0-3.75) vs. 4.5 (IQR 2-6), p=0.02) and the fact that the majority (8/16 (50%)) demonstrated a SOFA score of less than 3 and only 1/16 (6%) patient with a SOFA score >7 compared to 24/84 (29%) and 15/84 (18%) patients with NOD2 wild-type genotype, respectively (p=0.19). MBL deficiency (<0.5 µg/ml) had no influence on the severity overall as evaluated by the SOFA score (data not shown). However, severe MBL deficiency (<0.1 µg/ml) significantly increased the odds of a patient having severe organ dysfunction as defined by a SOFA score >7 (OR 5.57, 95% CI 1.25-24.85, p=0.02) after adjusting for age and gender. A similar non-significant trend was observed regarding severe MBL deficiency or *NOD2* mutations and frequency of admission to ICU (4/10 (40%) vs. 18/90 (20%) and 2/16 (13%) vs. 20-84 (24%), respectively), whereas in-hospital mortality was not different.

## Discussion

MBL and NOD2, two PRR of the innate immune system, have been implicated in the pathogenesis of *S. aureus* BSI in several experimental models [11–13,20,21]. This is the first human study designed to examine the effect of these two important first-line defense mechanisms on predisposition and severity of infection in *S. aureus* BSI patients, exclusively.

Despite previous experimental studies that were the basis for our *a priori* hypotheses, we did not demonstrate that MBL deficiency or *NOD2* mutations predispose to *S. aureus* BSI. Interestingly, we found that rather the opposite is true in terms of MBL deficiency. MBL deficiency was associated with less than half the risk of acquiring *S. aureus* BSI. Previous studies which failed to demonstrate an effect of MBL deficiency [25,30] were likely underpowered as they had only examined a limited number of *S. aureus BSI* patients as part of larger sepsis trials, and controls were not matched. Recent data may help to resolve this apparent contradiction. It has been demonstrated that wildtype *MBL2* genotypes are associated with persistent *S. aureus* nasal carriage in adults, a well known predictor for subsequent invasive disease [31]. Additionally, studies suggest that binding of MBL to *S. aureus* might be restricted to infancy due to inhibitory anti-wall teichoic acid antibodies in adults [23] and subsequently that anti-staphylococcal complement activation and opsonophagocytosis is dominated by the C1q-dependent classical pathway independent of MBL [22]. Finally, data that suggest a non-redundant role of MBL in staphylococcal infections in infancy come from two recent clinical studies that show that infants with *MBL2* mutations are more susceptible to *S. aureus* colonization [32] and fatal invasive methicillin-resistant *S. aureus* co-infections after influenza [33].

It is unlikely that an acute phase elevation of MBL accounts for the higher levels in cases as the degree of elevation is usually mild and restricted to wild-type patients [34], and more importantly, genotypic data of our case patients were consistent with MBL levels demonstrating high producing haplotypes in a significantly greater proportion compared to controls (61 vs. 39%).

We also found no difference in the prevalence of *NOD2* mutations in *S. aureus* BSI cases and controls with the frequency and presence of only heterozygous mutations being in line with previous reports from an Australian control population [35]. This finding is consistent with the clinical observation (and preliminary evidence [36]) that (untreated) Crohn’s disease patients, who have a higher prevalence of *NOD2* mutations than healthy controls, are not at an increased risk of *S. aureus* BSI.

Overall then it seems that physical factors (hemodialysis or intravenous catheters) or comorbidities (liver failure) account more for susceptibility to *S. aureus* BSI than genetic defects in the innate immune proteins we studies, at least in an adult population.

Although MBL deficiency or *NOD2* mutations had no significant impact on mortality, we could demonstrate important associations with our other *a priori* measure, the severity of *S. aureus* BSI as evaluated by the SOFA score. Interestingly, patients with *NOD2* mutations had significantly lower SOFA scores and admissions to ICU with 50% showing a SOFA score <3 as compared to only 29% of patients lacking tested *NOD2* polymorphisms. Less pulmonary inflammation and faster recovery has been shown in *NOD2* knockout mice during *S. aureus* pneumonia [13]. Similarly, a diminished initial inflammatory response was demonstrated in *NOD2* knockout mice after subcutaneous challenge with *S. aureus* [12] although the mice developed significantly larger ulcerations later on possibly related to an impaired bacterial clearance. In contrast, *NOD2* knockout mice were more susceptible to *S. aureus* infection in a peritoneal challenge model [11]. Currently available data on human sepsis associations with *NOD2* mutations indicate more prevalent bacteremia and higher sepsis-related mortality in ICU studies [14,15]. However, both human studies included only a minority of patients with *S. aureus* BSI, hence the ability to compare with our study is limited. In theory, heterozygous *NOD2* mutations might impair the recognition of *S. aureus* to a limited degree but also attenuate the initial excessive and dysfunctional inflammatory response [37,38]. In summary, this might effectively result in less host damage overall in *S. aureus* BSI assuming removal of the pathogen by timely administration of effective antibiotic treatment.

Only severe MBL deficiency (<0.1 µg/ml) was associated with critical disease as evaluated by the SOFA score and admission to ICU, which is in line with knockout animal models [20,21] and previous sepsis studies including a variety of infections [25,26,39]. However, the significance of this observation is limited by the small sample size of patients with severe MBL deficiency.

Our study has some limitations including the fact that microbial virulence factors shown to influence the severity of community-acquired invasive *S. aureus* infections recently [40] were not examined. In addition, data on *S. aureus* colonization rate, a recognized risk factor for invasive infections were not available in cases and controls. Severity according to the SOFA score was only evaluated once on the day the first positive blood culture was drawn before antibiotic therapy was initiated. However, this approach eliminates possible confounders introduced later by differences in treatment (e.g. antibiotic management, timing of surgical intervention or infectious diseases consultation). We limited our analysis of the innate immune system to two key PRR, which have been shown to be significantly involved in *S. aureus* infection, previously. Ideally, future studies should include other important PRR like TLR-2 [41] which are also involved in the pathogenesis of *S. aureus* infections. Although our analysis of the importance of MBL and NOD2 in *S. aureus* BSI is the largest to date, its significance is limited in terms of mortality due to low event numbers.

In conclusion, this study does not support an important role for either MBL or NOD2 in protecting adults from acquiring *S. aureus* BSI. In fact, contrary to previous animal model data our results show that MBL deficiency seems to confer significant protection from *S. aureus* BSI. In addition, heterozygous *NOD2* polymorphisms were less frequently associated with organ dysfunction in *S. aureus* BSI consistent with the notion that outcomes of infections are more driven by the host response to microorganisms than by their direct toxic effects [37,38]. Our present state of knowledge indicates that possible effects of innate immune system abnormalities are likely overwhelmed by conventional risks factors for staphylococcal BSI.

## Supporting Information

Table S1 **Taqman genotyping assay details (Life Technologies, Australia).**
(DOC)
