# Supplementary material for: Significance of Mannose-Binding Lectin Deficiency and Nucleotide-Binding Oligomerization Domain 2 Polymorphisms in Staphylococcus aureus Bloodstream Infections: A Case-Control Study
Source: PLoS One. 2013 Sep 27;8(9):e76218. doi: 10.1371/journal.pone.0076218 (PMC3785435; doi:10.1371/journal.pone.0076218)
Supplement: Table S1 — Taqman genotyping assay details (Life Technologies, Australia). (DOC) [file pone.0076218.s001.doc]

Table S1: Taqman genotyping assay details (Life Technologies, Australia)

| **Nucleotid Change** | **Localization** | **SNP Database ID** | **Assay Reference** | **Primers** | **Fluorescent Probes** |
| --- | --- | --- | --- | --- | --- |
| *MBL2* -X/Y | 10q11.2-q21 | rs7096206 | C__27858274_10 |  |  |
| *MBL2* -B (codon 54) | 10q11.2-q21 | rs1800450 | C___2336609_20 |  |  |
| *MBL2* –C (codon 57) | 10q11.2-q21 | rs1800451 | C___2336608_20 |  |  |
| *MBL2 -*D (codon 52) | 10q11.2-q21 | rs5030737 | C___2336610_10 |  |  |
|  |  |  |  |  |  |
| *NOD2* (R702W C>T) | 16q21 | rs2066844 | C__11717468_20 |  |  |
| *NOD2* (G908R G>C) | 16q21 | rs2066845 | C__11717466_20 |  |  |
| *NOD2* (L1007fsinsC -/C) | 16q21 | rs2066847 |  | 5'-GTCCAATAACTGCATCACCTACCT | VIC-CAGGCCCCTTGAAAG |
|  |  |  |  | 5'-CAGACTTCCAGGATGGTGTCATTC | FAM-CAGGCCCTTGAAAG |

Abbreviations: MBL, mannose-binding lectin; NOD2, nucleotide-binding oligomerization domain 2;

**Reference:**

1. Fishbein T, Novitskiy G, Mishra L, et al. NOD2-expressing bone marrow-derived cells appear to regulate epithelial innate immunity of the transplanted human small intestine. Gu**t 20**08; 57:323-30.
